# Neutrophil-to-lymphocyte and platelet-to-lymphocyte ratio help identify patients with lung cancer, but do not differentiate between lung cancer subtypes

**DOI:** 10.3325/cmj.2016.57.287

**Published:** 2016-06

**Authors:** Igor Nikolić, Suzana Kukulj, Miroslav Samaržija, Vjekoslav Jeleč, Marko Žarak, Biserka Orehovec, Ida Taradi, Dominik Romić, Toni Kolak, Leonardo Patrlj

**Affiliations:** 1 Department of Thoracic Surgery, Clinical Hospital “Dubrava,” Zagreb, Croatia; 2 Josip Juraj Strossmayer University of Osijek, Faculty of Medicine, Osijek, Croatia; 3 Clinic for Lung Diseases “Jordanovac,” University Hospital Centre Zagreb, Zagreb, Croatia; 4 Department of Neurosurgery, Clinical Hospital “Dubrava,” Zagreb, Croatia; 5 Clinical Department for Laboratory Diagnostics, Clinical Hospital “Dubrava,” Zagreb, Croatia; 6 Clinic for Surgery, Clinical Hospital “Dubrava,” Zagreb, Croatia

## Abstract

**Aim:**

To assess the diagnostic value of neutrophil-to-lymphocyte ratio (NLR) and platelet-to-lymphocyte ratio (PLR) in lung cancer (LC). We compared the ratios between healthy participants and all LC patients, as well patients with different pathohistological LC subtypes.

**Methods:**

We retrieved the data on neutrophil, lymphocyte, and platelet levels in 449 patients with different pathohistological LC subtypes (non-small cell LC, small-cell LC, atypical or metastatic LC, neuroendocrine, and sarcomatoid carcinoma) and 47 healthy controls. NLR and PLR were calculated by dividing the absolute number of neutrophils or platelets with the absolute number of lymphocytes.

**Results:**

There were significant differences in both NLR and PLR (*P* < 0.001) between all LC patients and the control group, but there were no differences between patients with different LC subtypes. Reciever operating characteristics analysis for NLR showed the optimal cut-off value of 2.71, with a sensitivity of 77.05% and specificity of 87.23%. The optimal cut-off value for PLR was 182.31, with a sensitivity of 51.09% and specificity of 91.49%.

**Conclusion:**

The results showed that the NLR and PLR may have added value in the early diagnosis of LC, but further research is needed to confirm these results.

Malignant diseases are among the most common causes of death (1), with lung carcinomas (LC) being the most diagnosed malignant disease and a leading cause of death from malignant disease in developed countries in 2012 (2).

Many of the carcinomas grow at the site of the infection, chronic irritation, and inflammation, and the most recent research shows that systemic inflammatory reaction plays a very important role in the development and spread of tumor cells. Secretion of different cytokines stimulated by inflammation induces angiogenesis and tumor invasion, also damaging the DNA. Tumor cells secrete different chemokines that attract neutrophils, monocytes, and lymphocytes. At the beginning of tumor growth, these cells create an environment that promotes growth and stimulates angiogenesis. Although inflammatory response should have an antitumor effect, in patients with developed tumors this response is changed (3). A recent study also has shown that leukocytes, neutrophils, alpha-1, and alpha-2 protein fractions are increased in non-small-cell lung cancer (4).

Neutrophils, the most numerous leukocytes in the peripheral blood, besides having a role in destroying tumor cells, also play an important role in tumor growth stimulation by secreting different cytokines, growth factors, proteases, and other molecules (5). On the other hand, lymphocytes protect the organism from tumor cells by blocking their proliferation and migration (2). Platelets, by secreting different growth factors, play an important role in inflammation, tissue regeneration, and immunologic response (6).

Although the therapy of LC has recently seen substantial advances, the early diagnostic accuracy of this condition remains unsatisfying. Therefore, in order to better diagnose LC patients, new markers are needed.

An imbalance between neutrophils and lymphocytes takes place due to hypoxia and necrosis caused by tumor cells, which is also connected to antiapoptosis. The studies so far (7-10) have found that NLR and PLR are good inflammatory response follow-up markers and predictive survival markers in patients with different carcinomas, including LC. Also, they can be easily obtained in everyday practice, without additional costs (11,12). However, their diagnostic value remained unexplained. This is why we decided to study diagnostic value of NLR and PLR in early diagnosis of different LC subtypes. Our hypothesis was that there was a difference in NLR and PLR between LC patients and healthy participants, and between patients with different LC subtypes. To the best of our knowledge, this research is the first to include all other pathohistological LC subtypes in the assessment of the diagnostic significance of NLR and PLR.

## Patients and methods

The absolute number of neutrophil granulocytes, lymphocytes, and platelets of LC patients diagnosed between January 2012 and December 2015 at the Clinic for Lung Diseases “Jordanovac,” University Hospital Centre Zagreb, and Clinical Hospital “Dubrava,” (n = 449) were retrieved from the hospital registries. We included only samples collected when LC was diagnosed for the first time, before any therapies (surgery, chemo- and radiotherapy) were initiated. The patients were divided into groups according to the pathohistologic cancer subtype: 1) small-cell LC (SCLC); 2) non-small cell LC (NSCLC); 3) atypical and metastatic LC; 4) neuroendocrine LC, and 5) sarcomatoid LC. Patients with NSCLC were further divided into: 2a) adenocarcinoma; 2b) planocellular carcinoma; 2c) non-classified carcinoma.

Controls were selected from the pool of employees of the Clinical Hospital “Dubrava” who underwent routine annual general medical examinations, and they were sex and age matched with the LC patients (n = 47). They had no history of any pulmonary or other diseases that could affect either NLR or PLR. The study was approved by the Ethics Committee of the Clinical Hospital “Dubrava.”

All blood samples were collected in vacutainer test tubes with potassium EDTA (kEDTA) as an anticoagulant. Differential blood counts (DBC) were performed at the Clinic for Lung Diseases “Jordanovac” using the hematology analyzer Coulter LH 750 (Beckman Coulter, Miami, FL, USA), and at the Clinical Hospital “Dubrava” using the Siemens ADVIA 2120i (Siemens Diagnostics, Tarrytown, NY, USA). NLR and PLR were calculated by dividing the absolute number of neutrophils or platelets with the absolute number of lymphocytes.

### Statistical analysis

The normality of the distribution was tested using the Kolmogorov-Smirnov test. NLR and PLR values are summarized as median with absolute range. Between-group differences were tested using the one-way analysis of variance (ANOVA) and Student-Newman-Keuls’s *post-hoc* test. Reciever operating characteristics (ROC) curves were used to calculate the optimal cut-off values for NLR and PLR to discriminate between LC patients and healthy participants, with an optimal proportion of false positive and false-negative results. All tests were two-sided and the significance level was set at *P* < 0.05. Statistical analyses were performed using MedCalc (MedCalc ver. 14, Mariakerke, Belgium).

## Results

The total number of patients who met the inclusion criteria was 449, but due to incomplete data, 61 were excluded, so the final number of analyzed patients was 388. Groups with neuroendocrine, sarcomatoid, metastatic, and atypical LC were not included in the statistical analysis due to a small number of participants. There were no differences in sex and age between cases and controls (289/388 [74.4%] men vs 37/47 [78.7%] men; 64 ± 9 years vs60 ± 6 years, respectively).

NLR and PLR values by groups are summarized in Table 1. ANOVA showed significant differences for both NLR and PLR (*P* < 0.001), and *post-hoc* differences were significant for all LC subtypes compared to the control group, while the differences between the LC subtypes were not significant (Table 1).

**Table 1:** Neutrophil-to-lymphocyte ratio (NLR) and platelet-to-lymphocyte ratio (PLR) values in lung carcinoma (LC) patients, divided into subtypes, and in the control group summarized as median with absolute range

Group	N (%)	NLR	PLR
Lung cancer			
small-cells LC (SCLC)*^†^	38 (9.8)	3.70 (1.15-23.07)	197.76 (16.36-464.29)
adenocarcinoma (NSCLC)*^†^	171 (44.1)	3.82 (1.04-24.75)	184.00 (30.70-637.04)
planocellular LC (NSCLC)*	120 (30.9)	3.67 (0.97-31.33)	171.63 (33.75-1006.67)
non-classified LC (NSCLC)*	37 (9.5)	4.71 (1.50-12.04)	206.43 (88.10-507.00)
metastatic or atypical LC	13 (3.4)	3.14 (1.64-11.47)	169.23 (76.43-313.33)
neuroendocrine LC	7 (1.8)	3.00 (2.31-8.80)	129.75 (108.46-421.10)
sarcomatoid LC	2 (0.5)	6.18, 7.10	170.59, 267.27
total	388 (100.0)	3.63 (0.97-31.33)	171 (16.36-1006.67)
Controls	47 (100.0)	2.07 (1.15-5.19)	115.00 (5.44-332.14)
*P* (ANOVA)		<0.001	<0.001

*Significantly different compared to controls.

†SCLC – small cells lung carcinoma; NSCLC – non-small cells lung carcinoma.

**Figure 1:**
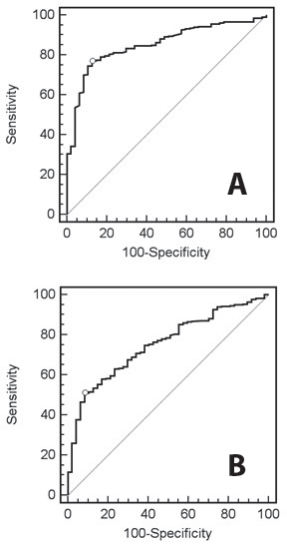
Receiver operating curve (ROC) for neutrophil-to-lymphocyte ratio (**A**) and platelet-to-lymphocyte ratio (**B**).

ROC analysis (Figure 1) was performed only for the NSCLC and SCLC subgroups, and other groups were excluded from the analysis due to the small number of patients (total n = 22). Area under the ROC curve for NLR was 0.852 (95% confidence interval: 0.814-0.885), with an optimal cut-off value of 2.71, sensitivity of 77.05%, and specificity of 87.23%. Area under the ROC curve for PLR was 0.753 (95% confidence interval: 0.709-0.794), with an optimal cut-off value of 182.31, sensitivity of 51.09%, and specificity of 91.49%.

If we combine the cut-off value for NLR and PLR together, there is 80.85% chance that a healthy individual will not have LC if both ratios are under the cut-off value and there is 78.96% chance that an LC patient will have any type of LC if one or both ratios are above the cut-off value.

## Discussion

Our research shows that NLR and PLR in patients with different pathohistologic LC subtypes are significantly higher than in controls. Also, this study showed that NLR and PLR have satisfying diagnostic value in diagnosis of LC.

These findings are consistent with similar previous research, which showed an association between inflammatory tumor growth progression and disease outcome (7-13). Inflammation contributes to the LC pathogenesis and development (3), and increased neutrophil infiltration with decreased lymphocyte infiltration in the tumor tissue is associated with a poor survival and response to therapy in some LC subtypes (8-12). In their meta-analyses, Peng et al (7) and Gun et al (8) showed an association between NLR and the clinical outcome of NSCLC. They showed that a high baseline NLR was associated with a poor overall survival and response to chemotherapy. Therefore, NLR could be useful in creating an individual patient therapy plan (7,8). Although DBC components are non-specific parameters for cancer diagnosis and prognosis, using their ratios (NLR and PLR) could be a new approach to early LC diagnostics.

Our research showed that there were no significant differences in NLR and PLR between patients with different LC subtypes, although such differences are expectable due to different invasiveness of the specific LC subtypes (14).

The evaluation of the diagnostic sensitivity and specificity of NLR and PLR using ROC analysis showed that the optimal NLR cutoff value was 2.71, and the optimal PLR cutoff value was 182.31. The area under the ROC curve showed that the cutoff NLR value (0.852) was more accurate in discriminating between LC patients and healthy controls than the PLR cutoff value (0.753). Better sensitivity of the NLR (77.05%) indicates that it is a better marker for the diagnosis of LC. Although sensitivity and specificity of both markers are quite satisfying, by combining them, and including other known markers, we could increase the sensitivity and specificity of the LC diagnostic process.

Increased PLR is a direct consequence of the increased platelet number, due to their possible role in tumor growth through the mechanisms of immunomodulation and angiogenesis (3). Platelets stimulate tumor growth by reinforcing angiogenesis via cytokines, the vascular endothelial growth factor, and platelet-derived growth factor. Tumors, by secreting factors that retain platelets, protect the environment that positively affects their survival (15). Through an interaction with the aforementioned platelet factors, tumors stimulate migration, proliferation, and epithelio-mesenchymal transition of other cells (10).

Neutrophils can promote tumor growth and metastasis by inhibiting the function of the cytotoxic lymphocytes and remodeling the tumor extracellular matrix. The number of neutrophils also increases with an increase in the number of tumor cells (6). Therefore, it is logical to expect an increased NLR, which is a systemic inflammatory index, in LC patients compared to the healthy population. In LC patients, the NLR reflects an imbalance in the pro- and anti-tumor activities of the immunologic response (16).

Lymphocytes play an important role in tumor protection by decreasing tumor-cell proliferation and migration. However, an increase in the neutrophil count, as an organism`s response to tumor presence, diminishes the cytolytic activity of lymphocytes and natural killer cells and suppresses the proliferation of T-cells (17). Neutrophil presence in the tumor micro-surroundings directly increases its survival, which has a negative impact on the organism (10).

Our research, conducted on a relatively large number of participants, and with an appropriate referent population, confirmed the results of previous research that showed an association between the inflammatory reaction and LC (18). Although previous studies assessed the association between the NLR and PLR with the disease prognosis, therapy outcome, and response to therapy in NSCLC patients, our research was the first to include all other pathohistological LC subtypes in the assessment of the diagnostic significance of NLR and PLR. The cutoff values for NLR>2.71 and PLR>182.31 could be considered in the future when diagnosing LC. These ratios have the advantage of being easily obtainable without additional costs during the initial patient assessment when a certain disease is suspected, which is important for the early diagnosis of LC. Results of the current research show that there is a significant difference in NLR and PLR between LC patients and healthy patients, but not between patients with different LC subtypes, which all points to the fact that NLR and PLR could be used only as general markers of the occurrence and development of LC, but not as markers of the occurrence and development of certain LC subtypes.

Although this study showed a good diagnostic value of NLR and PLR in LC diagnosis, which is a novelty in this field, it has some limitations. First, the patient inclusion criteria did not involve the stage of LC but only patohistologycal type. Also, we did not compare these data with the group of patients with other malignant diseases or similar conditions. Finally, different methodology in determination of DBC was used.

Future research on the diagnostic specificity and sensitivity of NLR and PLR should be made in combination with current LC markers, such as the cytokeratin fragment 21-1, neuron specific enolase, and alpha fetoprotein. It is possible that combination of the current markers could lead to a greater diagnostic sensitivity and specificity.
